# Global Real-World Evidence of Sofosbuvir/Velpatasvir as a Highly Effective Treatment and Elimination Tool in People with Hepatitis C Infection Experiencing Mental Health Disorders

**DOI:** 10.3390/v14112493

**Published:** 2022-11-11

**Authors:** Heiner Wedemeyer, Vito Di Marco, Montserrat Garcia-Retortillo, Elisabetta Teti, Chris Fraser, Luis Enrique Morano Amado, Sergio Rodriguez-Tajes, Silvia Acosta-López, Joss O’Loan, Michele Milella, Maria Buti, María Fernanda Guerra-Veloz, Alnoor Ramji, Mary Fenech, Alexandra Martins, Sergio M. Borgia, Kim Vanstraelen, Michael Mertens, Cándido Hernández, Ioanna Ntalla, Heribert Ramroth, Scott Milligan

**Affiliations:** 1 Department of Gastroenterology, Hepatology and Endocrinology, Hannover Medical School, OE6810, Carl-Neuberg-Str. 1, 30625 Hannover, Germany; 2 University of Palermo, Piazza Marina, 61, 90133 Palermo, Italy; 3 Liver Section, Gastroenterology Department, Hospital del Mar-Parc de Salut Mar, Hospital del Mar Medical Research Institute (IMIM), C/ del Dr. Aiguader, 88, 08003 Barcelona, Spain; 4 Tor Vergata University, Via Cracovia, 50, 00133 Rome, Italy; 5 Cool Aid Community Health Centre, 713 Johnson St, Victoria, BC V8W 1M8, Canada; 6 Unit of Infectious Diseases, Álvaro Cunqueiro University Hospital, Estrada de Clara Campoamor, 341, 36312 Vigo, Spain; 7 Liver Unit, Hospital Clinic Barcelona, IDIBAPS, CIBERehd, C. de Villarroel, 170, 08036 Barcelona, Spain; 8 Digestive Diseases, Hospital Nuestra Señora de Candelaria, Ctra. Gral. del Rosario, 145, 38010 Tenerife, Spain; 9 Medeco Inala & Kombi Clinic, 55b/156 Inala Ave, Brisbane, QLD 4077, Australia; 10 School of Medicine, University of Queensland, St Lucia, Brisbane, QLD 4072, Australia; 11 Clinic of Infectious Diseases, University of Bari, Piazza Umberto I, 1, 70121 Bari, Italy; 12 Liver Unit, Vall d’Hebron University Hospital, and CIBEREHD del Instituto Carlos III, Barcelona, Spain; 13 Virgen Macarena University Hospital, Av. Dr. Fedriani, 3, 41003 Seville, Spain; 14 Clinical Research Fellow in Hepatology at King’s College Hospital, Denmark Hill, London SE5 9RS, UK; 15 University of British Columbia, Vancouver, BC V6T 1Z4, Canada; 16 Queensland Injectors Health Network (QuIHN), Treatment and Management Programme, 1 Hamilton Pl, Bowen Hills, Brisbane, QLD 4006, Australia; 17 Hospital Prof. Dr. Fernando Fonseca, IC19, 2720-276 Amadora, Portugal; 18 Infectious Diseases, William Osler Health System, 2100 Bovaird Dr E, Brampton, ON L6R 3J7, Canada; 19 Gilead Sciences Europe Ltd., Stockley Park, 2 Roundwood Ave, Hayes, Uxbridge UB11 1AS, UK; 20 Trio Health Analytics, 1025 Cannon Street, Suite 2C, Louisville, CO 80027, USA

**Keywords:** HCV elimination, HCV, mental health disorders, real-world, sofosbuvir/velpatasvir

## Abstract

Hepatitis C virus (HCV) is prevalent in people with mental health disorders, a priority population to diagnose and cure in order to achieve HCV elimination. This integrated analysis pooled data from 20 cohorts in seven countries to evaluate the real-world effectiveness of the pangenotypic direct-acting antiviral (DAA) sofosbuvir/velpatasvir (SOF/VEL) in people with mental health disorders. HCV-infected patients diagnosed with mental health disorders who were treated with SOF/VEL for 12 weeks without ribavirin as part of routine clinical practice were included. The primary outcome was sustained virological response (SVR) in the effectiveness population (EP), defined as patients with an available SVR assessment. Secondary outcomes were reasons for not achieving SVR, characteristics of patients with non-virological failures, adherence, and time from HCV RNA diagnosis to SOF/VEL treatment initiation. A total of 1209 patients were included; 142 did not achieve an SVR for non-virological reasons (*n* = 112; 83 lost to follow-up, 20 early treatment discontinuations) or unknown reasons (*n* = 30). Of the 1067 patients in the EP, 97.4% achieved SVR. SVR rates in the EP were ≥95% when stratified by type of mental health disorder and other complicating baseline characteristics, including active injection drug use and antipsychotic drug use. Of 461 patients with data available in the EP, only 2% had an adherence level < 90% and 1% had an adherence level < 80%; all achieved SVR. Patients with mental health disorders can be cured of HCV using a well-tolerated, pangenotypic, protease inhibitor-free SOF/VEL regimen. This DAA allows the implementation of a simple treatment algorithm, with minimal monitoring requirements and fewer interactions with central nervous system drugs compared with protease-inhibitor DAA regimens.

## 1. Introduction

Hepatitis C virus (HCV) infection is a major global health concern with significant individual, societal, and economic impacts. The World Health Organization (WHO) has set the goal of eliminating viral hepatitis as a major public health threat by 2030 [1,2]. The achievement of this target has been met by many challenges, but the need to overcome these challenges in a timely manner is crucial. Data modelling the global impact of COVID-19 on HCV elimination efforts suggest that a single year of delay in hepatitis elimination programmes has the potential to result in tens of thousands of additional liver cancers and deaths from HCV globally [3]. Now is an important time to prioritise HCV care, with international guidelines recognising the need for improved access to care and cure in various priority populations to achieve HCV elimination [4].

One such population is made up of those with mental health disorders. The prevalence of psychiatric comorbidities is high in people with chronic HCV infection compared with the general population [5]. In the USA, 67.1% of adults with HCV have been reported to have mental health symptoms [6], and studies in Europe, North America and Australia have reported the prevalence of HCV in patients with severe mental illness to be between 4.6% and 17.4% [5,7,8,9,10].

Historically, there has been reluctance to treat HCV in patients with comorbid mental health disorders, largely due to the established association between interferon-based antiviral therapy and significant psychiatric side effects, and perceived poor compliance [5,11]. However, current direct-acting antivirals (DAAs) are clinically effective (≥95% cure rates), with favourable safety and tolerability profiles, low chance of late relapse following sustained virological response (SVR) [4,12], and no negative effects on mental health [11,13,14,15]. Psychoactive drugs, including antipsychotics such as quetiapine, are commonly prescribed in patients with mental health disorders [16,17,18]. Therefore, drug–drug interactions (DDIs) are an important consideration in the management of HCV in people with mental health disorders. DAAs and psychoactive drugs are extensively metabolised in the liver and can affect the activity of drug-metabolising enzymes such as CYP450, leaving patients at risk of increased comedication exposure and adverse events [18,19]. DAAs such as sofosbuvir/velpatasvir (SOF/VEL) do not show DDIs with most antipsychotics or any antidepressants [4,19]. These arguments should dismiss the negative historical perceptions around treating HCV in individuals with mental health disorders and encourage prompt treatment to support HCV elimination.

SOF/VEL is a pangenotypic, panfibrotic, protease inhibitor-free, once-daily, single-tablet regimen that can be taken with or without food and can be used for a fixed 12-week treatment duration in all adult patients with chronic HCV, with limited need for pre-treatment or on-treatment monitoring. These attributes support rapid treatment initiation after diagnosis, as recommended by international guidelines, particularly for those acknowledged to be less engaged in healthcare, including people with mental health disorders [4]. High rates of SVR across all HCV genotypes, in patients with or without compensated cirrhosis and irrespective of human immunodeficiency virus (HIV) status or previous treatment failure with interferon, ribavirin or protease inhibitors, have been reported in clinical trials of SOF/VEL [20,21,22,23]. Similar findings have been reported in real-world cohort studies across a range of clinical settings worldwide, including a large analysis of over 5000 patients [24,25].

This integrated data analysis pooled data from 20 clinical cohorts across seven countries (Australia, Canada, Germany, Italy, Portugal, Spain, USA) to evaluate the real-world effectiveness of a 12-week SOF/VEL regimen in people with mental health disorders.

## 2. Materials and Methods

### 2.1. Study Design

This analysis included adult patients diagnosed with mental health disorders, under care in various settings. Some of the settings of care included were tertiary liver clinics, community health centres, community outreach services, infectious diseases clinics, and addiction and harm reduction centres. Mental health disorders were evaluated according to standardised local procedures (e.g., ICD-9, ICD-10), determined from medical history and medications, or defined at a physician’s discretion. Individuals infected with HCV genotype 1–6, with or without compensated cirrhosis, who initiated treatment with a 12-week SOF/VEL 400/100 mg regimen (without ribavirin) as part of routine clinical practice, were eligible for inclusion. The full study methodology is available in previously published retrospective analyses of the effectiveness of SOF/VEL in other special populations (homeless and prison populations) [26,27]. Patients were managed and treated according to local guidelines and standards of care. Adherence was assessed by the treating physician according to the number of pills taken. Patients were categorised with an adherence level of ≥90%, <90%, ≥80%, or <80%. This retrospective analysis was based on the secondary use of data that were previously collected as part of routine clinical care and anonymised prior to analysis.

### 2.2. Outcomes

Effectiveness, defined as SVR 12 or 24 weeks after the end of treatment, was assessed in two populations. The overall population (OP) comprised all patients, including those with a virological, non-virological, or unknown reason for not achieving SVR. The effectiveness population (EP) included patients with an available SVR assessment and excluded patients with a non-virological or unknown reason for not achieving SVR. Non-virological reasons for not achieving SVR were defined as early treatment discontinuation, non-adherence (where associated with a lack of SVR assessment), reinfection, loss to follow-up (LTFU), death before SVR assessment, and consent withdrawal. Virological reasons for not achieving SVR were specified as virological breakthrough, non-response, or relapse, in cases where this level of detail was available.

The primary outcome was SVR in the EP overall, and stratified by mental health history, antipsychotic drug use, and injection drug use (IDU). Secondary outcomes were reasons for not achieving SVR, characteristics of patients with non-virological failures, adherence, and time from HCV RNA diagnosis to SOF/VEL treatment initiation.

### 2.3. Statistical Analyses

Descriptive characteristics were presented as the number (*n*) and percentage of patients (%) for the categorical variables. Continuous variables were summarised as mean (standard deviation; SD). Data were evaluated using descriptive statistics in R version 3.5.2.

## 3. Results

**Figure 1:**
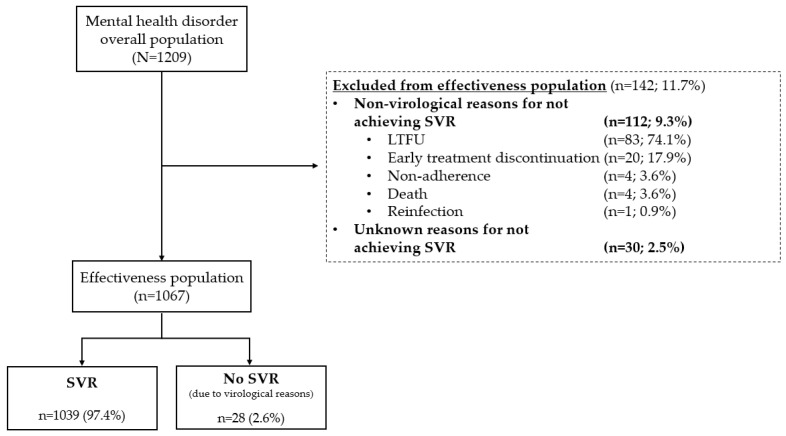
Flowchart of patients included in this real-world analysis. Effectiveness population includes all patients with a valid SVR12/24 result available. Abbreviations: LTFU: loss to follow up; SVR: sustained virological response.

A total of 1209 patients with HCV infection and mental health disorders were treated with SOF/VEL and comprised the OP in this integrated real-world analysis. A summary of patient disposition is shown in Figure 1, and patient baseline characteristics are shown in Table 1. Depression, anxiety and cognitive or psychiatric disorder were the most commonly reported mental health disorders, with 24.7% (278/1125) of patients with available data having two or more coexisting conditions. In 1010 patients where information on antipsychotic drug use was available, 35.3% (357/1010) received treatment with antipsychotics, predominantly quetiapine (*n* = 133). Where status was known, 55% (545/991) had a history of IDU; 21.1% (209/991) were still actively injecting drugs. The EP included 1067 patients, after exclusion of patients who did not achieve SVR due to non-virological (*n* = 112) or unknown reasons (*n* = 30).

### 3.1. Effectiveness

**Figure 2:**
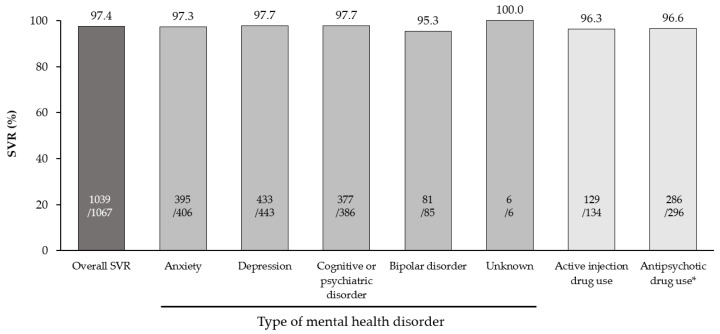
SVR in the effectiveness population, stratified by type of mental health disorder and complicating baseline characteristics. * For 21 patients in the EP, aggregated data for SVR were provided, without breakdown for anti-psychotic drug use. Abbreviations: EP: effectiveness population; SVR: sustained virological response.

In the overall EP, SVR was 97.4% (1039/1067), with SVR rates ≥ 95.3%, irrespective of the specific mental health disorder, or the presence of baseline factors that could complicate HCV cure, including active IDU and antipsychotic drug use (Figure 2). SVR was achieved in 100% of patients who received treatment within 1 day (27/27) or 1 week (49/49) of diagnosis, in 97.6% (452/463) of patients who received treatment within 90 days and in 95.6% (284/297) of patients who received treatment more than 90 days after diagnosis. Adherence data were available for 461 patients in the EP. Of this group, 2.2% (10/461) of patients had an adherence level < 90% and 1.1% (5/461) had an adherence level <80%; all achieved SVR. In patients where the adherence level was unknown, 96.9% (587/606) achieved SVR. Virological reasons for not achieving SVR were reported for 2.6% (28/1067) of patients, predominantly due to relapse (25/28).

### 3.2. Non-Virological and Unknown Reasons for Not Achieving SVR

In the OP, the predominant reasons for not achieving SVR were non-virological (9.3%; 112/1209), with 74.1% of these (83 patients) LTFU after treatment completion. Other non-virological reasons were early treatment discontinuation (17.9%; 20/112), non-adherence (3.6%; 4/112), reinfection (0.9%; 1/112) and death (3.6%; 4/112). Table 2 describes the baseline characteristics of patients who experienced a non-virological failure, including those who were LTFU. Unknown reasons were reported for 2.5% (30/1209) of patients.

## 4. Discussion

In this large integrated real-world data analysis, high cure rates were achieved with a regimen of SOF/VEL for 12 weeks in people experiencing mental health disorders, supporting the journey towards HCV elimination by addressing high-priority populations with HCV. These findings are in line with cure rates in more general populations in both clinical trials and real-world settings [20,21,24]. Cure rates in this analysis were high, irrespective of type of mental disorder, and despite the presence of factors historically considered complications for HCV cure, such as injection drug and antipsychotic use. This analysis adds to the growing body of evidence for the effectiveness of DAAs in HCV patients with mental health disorders [28]. This is the largest analysis to date of the effectiveness of SOF/VEL in this priority population, and results are in line with those reported in a smaller retrospective chart review in 579 patients with HCV and a mental health disorder, substance use, or both [28]. Moreover, in the current analysis, data on specific antipsychotic medications used, medication adherence, and time-to-treatment were captured.

In line with the established, favourable safety and tolerability profile of SOF/VEL as a protease inhibitor-free DAA, only 20 individuals discontinued SOF/VEL treatment early, and an adherence level ≥ 90% was reported in 512 patients (of 530 patients with adherence information available in the OP) [20,21,24]. Moreover, the low rates of non-adherence seen in this analysis with a simple SOF/VEL regimen (only 11 patients with <80% adherence in the OP) can address the historical reluctance to treat HCV patients with comorbid mental health disorders.

The question that often remains in the journey towards HCV elimination is why certain patients do not reach SVR due to non-virological reasons, including LTFU. In the low number of patients not achieving SVR in this current analysis, the main reason for not achieving SVR was LTFU. Table 2 demonstrates that reasons for LTFU are likely to be multifactorial, as shown previously in other priority populations, such as in those who use or inject drugs [29,30,31], making it difficult to predict who might be at risk of non-virological failure. However, as data emerge supporting flexibility in the timing of SVR assessment [32,33], and guidelines suggest that SVR assessment can be omitted altogether in certain patients [4], LTFU rates could become less clinically important when simplification of the HCV pathway and treatment at a population level is the focus to achieve HCV elimination. Moreover, as all LTFU in this analysis occurred after treatment completion, it is likely that most of these patients would have been cured given the large body of evidence about high SVR rates achieved with SOF/VEL.

One important consideration in treating patients with HCV and mental health disorders is the potential for DDIs, since co-administration of DAAs and central nervous system (CNS) drugs is common. In this real-world analysis, around a third of patients with data available were receiving antipsychotics during SOF/VEL treatment (Table 1), which is in line with recent findings in several European cohorts of patients receiving pangenotypic DAAs where CNS drug use was reported [17,34,35]. Results from previous real-world studies have shown that DDI risk is higher in patients treated with glecaprevir/pibrentasvir (GLE/PIB) versus SOF/VEL, for patients receiving antipsychotics [17,35]. The use of simple DAA regimens requiring minimal monitoring not only offers the possibility of treating all HCV patients with mental health disorders but also enables the opportunity for a rapid treatment start after diagnosis and implementation of test-and-treat strategies, through decentralisation of care and task-sharing to non-specialists, including mental health providers, as supported by the international AASLD/ALEH/APASL/EASL joint call to action [36]. This real-world analysis demonstrates that rapid treatment start is feasible in patients with mental health disorders, as all patients who were treated within 1 week or even 1 day of diagnosis achieved SVR. However, it also demonstrates that further action is needed to implement test-and-treat strategies and rapid treatment initiation in all patients, as treatment was initiated after more than 90 days in 39% of patients.

This analysis has the usual limitations associated with retrospective real-world data analyses. As this analysis included multiple sites with different recording strategies, not all data were available for all patients. However, overall SVR remained high despite missing values for characteristics such as cirrhosis status, genotype, IDU and type of mental health disorder, minimising the impact of these missing data. The characteristics of patients demonstrate that a diverse population was included in this analysis, minimising the concern of selection bias. Finally, DDI information for CNS drugs was not collected; however, few clinically relevant interactions would have been expected for SOF/VEL with these drugs, based on international treatment guidelines and the University of Liverpool Hep Drug Interaction Checker [4,19].

As this analysis was conducted in cohorts from developed countries, whether the findings are translatable to developing countries could be considered a limitation. However, a recent study in a diverse population of 399 patients with HCV from high-, middle- and low-income settings (Brazil, South Africa, Thailand, Uganda, and the USA) has demonstrated the effectiveness and feasibility of a minimal monitoring approach with SOF/VEL [37].

## 5. Conclusions

This large real-world analysis demonstrates that HCV can successfully be cured in the high-priority population of patients with mental health disorders, using a simple, well-tolerated, pangenotypic, protease inhibitor-free SOF/VEL regimen. SOF/VEL supports advances towards HCV elimination by allowing the implementation of a simplified treatment algorithm, with minimal monitoring requirements and fewer DDIs with CNS drugs compared with protease-inhibitor DAA regimens, therefore enabling a test-and-treat strategy and rapid treatment start, irrespective of the clinical setting.

## Figures and Tables

**Table 1:** Baseline demographics and clinical characteristics.

Characteristics	Overall Population (*N* = 1209)	Effectiveness Population (*n* = 1067)
Age, years, mean (SD) *	53.4 (13.4)	54.2 (13.7)
Sex, male, *n* (%)	730 (60.4)	595 (55.8)
Fibrosis stage, *n* (%)		
F0–F2	647 (53.5)	559 (52.4)
F3	169 (14.0)	151 (14.2)
F4	261 (21.6)	202 (18.9)
Unknown	132 (10.9)	155 (14.5)
Treatment history, *n* (%)		
Treatment-naïve	1047 (86.6)	920 (86.2)
Treatment-experienced (DAA-naïve)	162 (13.4)	147 (13.8)
HCV, *n* (%)		
GT1	532 (44.0)	482 (45.2)
GT2	199 (16.5)	185 (17.3)
GT3	385 (31.8)	319 (29.9)
GT4–6	69 (5.7)	61 (5.7)
GT mixed/unknown	24 (2.0)	20 (1.9)
Injection drug use, former or current, *n* (%)		
Yes	545 (45.1)	424 (39.7)
Active drug use, *n* (%)	209 (17.3)	134 (12.6)
No	446 (36.9)	386 (36.2)
Unknown	218 (18.0)	257 (24.1)
Type of mental health disorder ^†^, *n* (%)		
Anxiety	467 (38.6)	406 (38.1)
Depression	505 (41.8)	443 (41.5)
Bipolar disorder	99 (8.2)	85 (8.0)
Cognitive or psychiatric disorder	440 (36.4)	386 (36.2)
Unspecified ^‡^	7 (0.6)	6 (0.6)
Number of specified mental health disorders ^§^		
1	847 (70.1)	764 (71.6)
2	249 (20.6)	207 (19.4)
3	29 (2.4)	25 (2.3)
Use of 1 or more antipsychotic drugs, *n* (%)		
Yes	357 (29.5)	317 (29.7)
Quetiapine ^‖^	133 (11)	116 (10.9)
Aripiprazole ^‖^	41 (3.4)	34 (3.2)
Clozapine ^‖^	26 (2.2)	22 (2.1)
Paliperidone ^‖^	27 (2.2)	26 (2.4)
No	653 (54.0)	575 (53.9)
Unknown	199 (16.5)	175 (16.4)
Adherence ^¶^		
≥90%	512 (96.6)	451 (97.8)
<90%	18 (3.4)	10 (2.2)
≥80%	519 (97.9)	456 (98.9)
<80%	11 (2.1)	5 (1.1)
Time from HCV RNA diagnosis to SOF/VEL treatment start, mean (SD), days **	156 (607)	152 (625)
Time from HCV RNA diagnosis to SOF/VEL treatment start, days, *n* (%) ^#^		
<1	30 (3.6)	27 (3.6)
≤7	54 (6.5)	49 (6.4)
≤30	195 (23.3)	182 (23.9)
≤90	510 (60.9)	463 (60.9)
>90	327 (39.1)	297 (39.1)
Unknown	372 (30.8)	307 (28.8)

* Data available for 1120 patients in the OP and 994 patients in the EP. ^†^ Including patients for whom information on the specific mental health disorder was reported (OP: *n* = 1202; EP: *n* = 1061). Overlap between mental health disorders was possible. ^‡^ Patients flagged as having a mental health disorder where a specific disorder was not reported. ^§^ Number of mental disorder categories which the patient belongs to; e.g., a patient with paranoia and dementia will belong to one category (cognitive or psychiatric disorder), while a patient with anxiety and depression will belong to two. ^‖^ These medications are specifically selected as they were of interest; however, others are included. ^¶^ Percentage calculated using the number of patients with adherence information available as the denominator (OP: *n* = 530; EP: *n* = 461). ^#^ Percentage calculated using patients with time-to-treatment data available as denominator (OP: *n* = 837; EP: *n* = 760). ** Data available for 837 patients in the OP and 760 patients in the EP. Abbreviations: DAA: direct-acting antiviral; EP: effectiveness population; GT: genotype; OP: overall population; SD: standard deviation; SOF/VEL: sofosbuvir/velpatasvir.

**Table 2:** Baseline demographic characteristics of patients who did not achieve SVR due to non-virological reasons, including those LTFU.

Characteristics	Non-Virological Failures (*n* = 112) *	Patients LTFU (*n* = 83) ^†^
Sex, male, *n* (%)	69 (61.6)	54 (65.1)
Fibrosis stage, *n* (%)		
F0–F2	64 (57.1)	54 (65.1)
F3	11 (9.8)	8 (9.6)
F4	17 (15.2)	9 (10.8)
Unknown	20 (17.9)	12 (14.5)
Treatment history, *n* (%)		
Treatment-naïve	93 (83.0)	70 (84.3)
Treatment-experienced (DAA-naïve)	7 (6.2)	7 (8.4)
HCV, *n* (%)		
GT1	41 (36.6)	34 (41.0)
GT2	11 (9.8)	6 (7.2)
GT3	45 (40.2)	35 (42.2)
GT4–6	6 (5.4)	5 (6.0)
GT mixed/unknown	9 (8.0)	3 (3.6)
Injection drug use, former or current, *n* (%)		
Yes	72 (64.3)	63 (75.9)
Active drug use, *n* (%)	43 (38.4)	36 (43.4)
No	9 (8.0)	7 (8.4)
Unknown	31 (27.7)	13 (15.7)
Use of 1 or more antipsychotic drugs, *n* (%)		
Yes	28 (25.0)	21 (25.3)
No	48 (42.9)	35 (42.2)
Unknown	36 (32.1)	27 (32.5)

* A total of 83 (74.1%) LTFU, reinfection in 1 (0.9%), early discontinuation in 20 (17.9%), non-adherence in 4 (3.6%) and death in 4 (3.6%). ^†^ LTFU is a subset of non-virological reasons for not achieving SVR. Abbreviations: DAA: direct-acting antiviral; GT: genotype; LTFU: loss to follow up; SVR: sustained virological response.

## Data Availability

Data are contained within the article.

## References

[1] World Health Organization (2016). Global Health Sector Strategy on Viral Hepatitis 2016–2021. Towards Ending Viral Hepatitis. https://apps.who.int/iris/bitstream/handle/10665/246177/WHO-HIV-2016.06-eng.pdf?sequence=1.

[2] World Health Organization (2021). Interim Guidance for Country Validation of Viral Hepatitis Elimination. https://www.who.int/publications/i/item/9789240028395.

[3] Blach S., Kondili L.A., Aghemo A., Cai Z., Dugan E., Estes C., Gamkrelidze I., Ma S., Pawlotsky J.-M., Razavi-Shearer D. (2021). Impact of COVID-19 on global HCV elimination efforts. J. Hepatol..

[4] Pawlotsky J.M., Negro F., Aghemo A., Berenguer M., Dalgard O., Dusheiko G., Marra F., Puoti M., Wedemeyer H., European Association for the Study of the Liver (2020). EASL recommendations on treatment of hepatitis C: Final update of the series. J. Hepatol..

[5] Schaefer M., Capuron L., Friebe A., Diez-Quevedo C., Robaeys G., Neri S., Foster G.R., Kautz A., Forton D., Pariante C.M. (2012). Hepatitis C infection, antiviral treatment and mental health: A European expert consensus statement. J. Hepatol..

[6] Williams S.A., Lindley L.C. (2020). Characteristics of adults with hepatitis C virus: Evidence from the National Health and Nutrition Examination Survey 2011–2012. Gastroenterol. Nurs..

[7] Hughes E., Bassi S., Gilbody S., Bland M., Martin F. (2016). Prevalence of HIV, hepatitis B, and hepatitis C in people with severe mental illness: A systematic review and meta-analysis. Lancet Psychiatry.

[8] Bauer-Staeb C., Jörgensen L., Lewis G., Dalman C., Osborn D.P.J., Hayes J.F. (2017). Prevalence and risk factors for HIV, hepatitis B, and hepatitis C in people with severe mental illness: A total population study of Sweden. Lancet Psychiatry.

[9] Lluch E., Miller B.J. (2019). Rates of hepatitis B and C in patients with schizophrenia: A meta-analysis. Gen. Hosp. Psychiatry.

[10] Braude M.R., Con D., Lubel J., Bidwai A., Nguyen H.-T., Sharmamiglani S., Clarke D., Dev A., Sievert W. (2021). Liver disease prevalence and severity in people with serious mental illness: A cross-sectional analysis using non-invasive diagnostic tools. Hepatol. Int..

[11] Sundberg I., Lannergård A., Ramklint M., Cunningham J.L. (2018). Direct-acting antiviral treatment in real world patients with hepatitis C not associated with psychiatric side effects: A prospective observational study. BMC Psychiatry..

[12] Ghany M.G., Morgan T.R. (2020). AASLD-IDSA Hepatitis C Guidance Panel. Hepatitis C Guidance 2019 Update: American Association for the Study of Liver Diseases-Infectious Diseases Society of America Recommendations for Testing, Managing, and Treating Hepatitis C Virus Infection. Hepatology.

[13] Sackey B., Shults J.G., Moore T.A., Rogers R., Mehvar M., King J.G. (2018). Evaluating psychiatric outcomes associated with direct-acting antiviral treatment in veterans with hepatitis C infection. Ment. Health Clin..

[14] Gallach M., Vergara M., da Costa J.P., Miquel M., Casas M., Sanchez-Delgado J., Dalmau B., Rudi N., Parra I., Monllor T. (2018). Impact of treatment with direct-acting antivirals on anxiety and depression in chronic hepatitis C. PLoS ONE.

[15] Jain M.K., Thamer M., Therapondos G., Shiffman M.L., Kshirsagar O., Clark C., Wong R.J. (2019). Has access to hepatitis C virus therapy changed for patients with mental health or substance use disorders in the direct-acting-antiviral period?. Hepatology.

[16] Davidson K., Boyle A., Barclay S., Boxall E., Fleming C., Gossman P., McAvennie J., Reilly E., Sheridan E., Sommerville A. The quetiapine question: Management strategies for drug-drug interactions with antipsychotics and direct acting antivirals; a multicentre review. Proceedings of the Digital International Liver Congress.

[17] Sicras-Mainar A., Morillo-Verdugo R. (2020). Concomitant use of direct-acting antivirals (DAA) and central nervous system drugs in patients with hepatitis C virus infection. Adicciones.

[18] Smolders E.J., de Kanter C.T.M.M, de Knegt R.J., van der Valk M., Drenth J.P.H., Burger D.M. (2016). Drug-drug interactions between direct-acting antivirals and psychoactive medications. Clin. Pharmacokinet..

[19] Liverpool HEP Interactions. https://www.hep-druginteractions.org/checker.

[20] Feld J.J., Jacobson I.M., Hézode C., Asselah T., Ruane P.J., Gruener N., Abergel A., Mangia A., Lai C.-L., Chan H.L.Y. (2015). Sofosbuvir and Velpatasvir for HCV Genotype 1, 2, 4, 5, and 6 Infection. N. Engl. J. Med..

[21] Foster G.R., Afdhal N., Roberts S.K., Bräu N., Gane E.J., Pianko S., Lawitz E., Thompson A., Shiffman M.L., Cooper C. (2015). Sofosbuvir and Velpatasvir for HCV Genotype 2 and 3 Infection. N. Engl. J. Med..

[22] Curry M.P., O’Leary J.G., Bzowej N., Muir A.J., Korenblat K.M., Fenkel J.M., Reddy K.R., Lawitz E., Flamm S.L., Schiano T. (2015). Sofosbuvir and Velpatasvir for HCV in Patients with Decompensated Cirrhosis. N. Engl. J. Med..

[23] Wyles D., Bräu N., Kottilil S., Daar E.S., Ruane P., Workowski K., Luetkemeyer A., Adeyemi O., Kim A.Y., Doehle B. (2017). Sofosbuvir and Velpatasvir for the Treatment of Hepatitis C Virus in Patients Coinfected With Human Immunodeficiency Virus Type 1: An Open-Label, Phase 3 Study. Clin. Infect. Dis..

[24] Mangia A., Milligan S., Khalili M., Fagiuoli S., Shafran S.D., Carrat F., Ouzan D., Papatheodoridis G., Ramji A., Borgia S.M. (2020). Global real-world evidence of sofosbuvir/velpatasvir as simple, effective HCV treatment: Analysis of 5552 patients from 12 cohorts. Liver Int..

[25] Cheng P.-N., Mo L.-R., Chen C.-T., Chen C.-Y., Huang C.-F., Kuo H.-T., Lo C.-C., Tseng K.-C., Huang Y.-H., Tai C.-M. (2021). Sofosbuvir/Velpatasvir for Hepatitis C Virus Infection: Real-World Effectiveness and Safety from a Nationwide Registry in Taiwan. Infect. Dis. Ther..

[26] Conway B., Rodriguez-Tajes S., Garcia-Retortillo M., Pérez-Hernandez P., Teti E., Ryan P., Fraser C., Macedo G., Amado L.E.M., de Lédinghen V. (2022). Real-world evidence of sofosbuvir/velpatasvir as an effective and simple hepatitis C virus treatment and elimination tool in homeless populations. Future Virol..

[27] Rosati S., Wong A., Di Marco V., Pérez-Hernandez P., Macedo G., Brixko C., Ranieri R., Campanale F., Basciá A., Fernández-Rodríguez C. (2022). Real-world effectiveness of sofosbuvir/velpatasvir for the treatment of hepatitis C virus in prison settings. Future Virol..

[28] Ifeachor A.P., Houck K.K., Schulte S., Ansara E., Johnson A.J., Carr T.A., Liangpunsakul S. (2020). HCV eradication in veterans with underlying mental health disorders and substance use. J. Am. Pharm. Assoc..

[29] Darvishian M., Wong S., Binka M., Yu A., Ramji A., Yoshida E.M., Wong J., Rossi C., Butt Z.A., Bartlett S. (2020). Loss to follow-up: A significant barrier in the treatment cascade with direct-acting therapies. J. Viral Hepat..

[30] Christensen S., Buggisch P., Mauss S., Böker K.H.W., Schott E., Klinker H., Zimmermann T., Weber B., Reimer J., Serfert Y. (2018). Direct-acting antiviral treatment of chronic HCV-infected patients on opioid substitution therapy: Still a concern in clinical practice?. Addiction.

[31] Christensen S., Buggisch P., Mauss S., Böker K.H.W., Müller T., Klinker H., Zimmermann T., Serfert Y., Weber B., Reimer J. (2019). Alcohol and Cannabis Consumption Does Not Diminish Cure Rates in a Real-World Cohort of Chronic Hepatitis C Virus Infected Patients on Opioid Substitution Therapy—Data From the German Hepatitis C-Registry (DHC-R). Subst. Abus. Res. Treat..

[32] Sulkowski M., Feld J., Reau N., Scherbakovsky S., Hernández C., Vanstraelen K., Hammond K., Kreter B., Suri V., Ni L. Concordance between SVR4, SVR12, and SVR24 in HCV-infected patients who received fixed-dose combination sofosbuvir/velpatasvir in Phase 3 clinical trials. Proceedings of the International Liver Congress.

[33] Gane E., de Ledinghen V., Dylla D.E., Rizzardini G., Shiffman M.L., Barclay S.T., Calleja J.L., Xue Z., Burroughs M., Gutierrez J.A. (2021). Positive predictive value of sustained virologic response 4 weeks posttreatment for achieving sustained virologic response 12 weeks posttreatment in patients receiving glecaprevir/pibrentasvir in Phase 2 and 3 clinical trials. J. Viral. Hepat..

[34] Mangia A., Scaglione F., Toniutto P., Pirisi M., Coppola N., Di Perri G., Nieto G.A., Calabrese S., Hernandez C., Perrone V. (2021). Drug–Drug Interactions in Italian Patients with Chronic Hepatitis C Treated with Pangenotypic Direct Acting Agents: Insights from a Real-World Study. Int. J. Environ. Res. Public Health.

[35] Fagiouli S., Milligan S., Turnés J., Mangia A., Hintz A., Wick N., Sicras A., Esposti LD., Tacke F., Morillo R. Multinational evaluation of comedication and drug–drug interactions in hepatitis C patients treated with pangenotypic direct-acting antivirals. Proceedings of the Global Hepatitis Summit.

[36] AASLD/EASL/APASL/ALEH (2019). Call to Action for Liver Associations to Advance Progress Towards Viral Hepatitis Elimination: A Focus on Simplified Approaches to HCV Testing and Cure. https://www.aasld.org/call-action-advance-progress-towards-viral-hepatitis-elimination.

[37] Solomon S.S., Wagner-Cardoso S., Smeaton L., Sowah L.A., Wimbish C., Robbins G., Brates I., Scello C., Son A., Avihingsanon A. (2022). A minimal monitoring approach for the treatment of hepatitis C virus infection (ACTG A5360 [MINMON]): A phase 4, open-label, single-arm trial. Lancet Gastroenterol. Hepatol..

